# Physicians’ Attitudes Toward Artificial Intelligence in Medicine: Mixed Methods Survey and Interview Study

**DOI:** 10.2196/74187

**Published:** 2025-08-26

**Authors:** Helen Heinrichs, Alexander Kies, Saskia K Nagel, Fabian Kiessling

**Affiliations:** 1 Institute for Experimental Molecular Imaging Rheinisch-Westfälische Technische Hochschule Aachen (RWTH Aachen University) Aachen Germany; 2 Service and Technology Marketing Rheinisch-Westfälische Technische Hochschule Aachen (RWTH Aachen University) Aachen Germany; 3 Applied Ethics Rheinisch-Westfälische Technische Hochschule Aachen (RWTH Aachen University) Aachen Germany; 4 Fraunhofer Institute for Digital Medicine MEVIS Bremen Germany

**Keywords:** artificial intelligence, AI, attitudes, health care, medical diagnostics, AI adoption, medical AI, medical ethics, mixed methods, survey, qualitative interview

## Abstract

**Background:**

Artificial intelligence (AI) has the potential to transform clinical practice and diagnostics. Amid workforce shortages, AI-based applications assist in decision-making, patient monitoring, and administrative tasks. However, despite enthusiasm, integration into clinical practice remains limited because of concerns about usability, ethical implications, and physicians’ acceptance. Understanding physicians’ attitudes and engaging them in AI development may foster acceptance and adoption.

**Objective:**

This study aimed to comprehensively assess physicians’ attitudes toward AI in medicine.

**Methods:**

We conducted a mixed methods study combining a web-based survey and qualitative interviews. The survey explored physicians’ perspectives on the advantages and disadvantages of AI, its role in decision-making, and impact on physician-patient communication. Attitudes were measured using a 5-point Likert scale, covering cognitive and affective dimensions. An exploratory factor analysis (EFA) identified underlying attitudinal factors, while the Mann-Whitney *U* and the Kruskal-Wallis tests examined differences in attitudes based on physicians’ age, discipline, AI familiarity, and other variables. Overall, 13 physicians, independent of the survey sample, participated in semistructured interviews, which were analyzed using inductive coding and thematic analysis.

**Results:**

The survey yielded 498 responses. EFA revealed two factors: (1) *AI enthusiasm and acceptance* (Cronbach α=0.83) and (2) *AI skepticism and apprehension* (Cronbach α=0.77). Physicians reported high AI enthusiasm (median 4, IQR 3.57-4.29) and lower skepticism (median 3.62, IQR 3.20-4.20; reverse coded, with higher scores indicating reduced skepticism). Greater AI familiarity, use in daily life or professionally, and involvement in research were strongly associated with greater enthusiasm and lower skepticism. Physicians involved in AI-related research reported significantly higher enthusiasm (mean rank: AI research=111.52; no AI research=54.32; *P*<.001) and lower skepticism (mean rank: AI research=108.27; no AI research=70.45; *P*=.01). Those using AI professionally or intending to do so similarly expressed high enthusiasm (mean rank: professional use=253.88; no use=196.17; *P*=.001) and lower skepticism (mean rank: plan to use=275.93; no use=218.86; *P*=.001). Greater familiarity with AI tools was also strongly associated with higher enthusiasm (mean rank: very familiar=323.55; not familiar=169.86; *P*<.001) and lower skepticism (mean rank: very familiar=296.90; not familiar=186.23; *P*=.008). Chief physicians (mean rank 277.32) were significantly less skeptical than residents (mean rank 210.60; *P*=.01); however, age and discipline did not influence attitudes. The qualitative analysis identified six themes shaping physicians’ attitudes: (1) status quo, (2) AI dependency and negligence, (3) role changes and needs, (4) AI transparency and decision-making, (5) the physician-patient relationship, and (6) a framework for responsible AI integration. These findings led to several key propositions considered critical for AI adoption.

**Conclusions:**

AI in medicine is viewed positively, with attitudes shaped more by experience and engagement than by demographic factors. While concerns persist, they diminish with increased familiarity and professional use. These findings highlight the need for targeted education, hands-on training, and standardized implementation strategies to enhance AI engagement and facilitate adoption.

## Introduction

The health care sector faces numerous challenges, including demographic changes such as an aging population, which amplify the prevalence and complexity of chronic diseases and, consequently, the demand for health care workers. Health care workers must adapt to these challenges amid persistent workforce shortages [1,2], with Europe alone facing an expected shortfall of 4 million health care workers by 2030 [3]. In this context, digital health technologies, particularly artificial intelligence (AI), are emerging as potential solutions to strengthen health care systems and meet growing demands.

AI-based technologies offer unprecedented opportunities to transform health care by enhancing efficiency, reducing costs, and alleviating the workload of physicians [2,4]. These technologies, which use self-learning algorithms to process large amounts of data and assist in decision-making with varying degrees of autonomy, can mimic some human cognitive functions [5]. The scope of AI applications in health care is diverse and promising, ranging from enhancing diagnostic accuracy to optimizing personalized treatments. For instance, AI can facilitate large-scale pancreatic cancer detection using noncontrast computed tomography and deep learning [6], enable breast cancer classification by combining radiomics and autoencoder features [7], and predict drug efficacy in cancer therapy through machine learning [8].

Recognizing the potential of AI, policy makers are promoting its responsible development and integration. A European Parliament study [9] highlights the need for multistakeholder collaboration among AI developers, clinical end users, and biomedical and ethical researchers. Such collaboration is critical for the adoption of AI, as outlined in the European AI Act, the world’s first comprehensive AI regulation, enacted in 2024 [10]. Similarly, Germany has launched the AI Action Plan to boost research and innovation; address demographic changes and workforce challenges; and fund research on the ethical, legal, and social implications of AI in health care [11].

Despite these initiatives, challenges such as data integrity, privacy and legal concerns, limited clinical validation, underdeveloped digital infrastructure, and inequities in access hinder the large-scale implementation and widespread adoption of AI [12,13]. For instance, in Germany, the telematics infrastructure and digitized hospital communication remain inadequate, indicating foundational shortcomings in the integration of AI [14,15]. If left unaddressed, these challenges may lead to ethical and social consequences, including a loss of trust in physicians [16].

The attitudes of physicians play a pivotal role in the successful adoption and integration of AI into clinical practice. However, perceived threats to professional autonomy [17] and limited familiarity with AI [18] hinder its acceptance. Therefore, actively engaging and acknowledging physicians as key users in research and development is crucial for creating practical and ethically sound applications [19]. Previous research has largely focused on specific AI applications, such as clinical decision support systems and chatbot services, often targeting specific physician disciplines, medical students [20,21], or patients [22,23]. Moreover, many studies rely on singular research methods, such as surveys or interviews, potentially limiting deeper insights [20].

To address this gap, we comprehensively studied the attitudes of physicians toward AI across diverse disciplines and experience levels, emphasizing their critical role in AI adoption. Our study integrated 3 complementary data sources to triangulate the findings: a quantitative survey investigating physicians’ attitudes and how these vary according to demographic and professional characteristics, an open-ended question capturing nuanced responses and novel insights, and qualitative interviews providing deeper context on physicians’ attitudes and expectations. The interviews further informed propositional statements and recommendations, which are valuable for guiding future research and policy making. By addressing this gap, we seek to align AI development with clinical needs, foster trust and acceptance among physicians, and enhance the effectiveness of AI in practice, ultimately improving patient care. Therefore, this study aims to comprehensively assess physicians’ attitudes and expectations toward AI adoption, using a mixed methods approach to identify key factors influencing their acceptance and skepticism, and to promote successful integration into clinical practice.

## Methods

### Study Design and Approach

This study used an explanatory sequential mixed methods approach [24], consisting of (1) standardized surveys; (2) an open-ended survey question, “Is there anything else you would like to add to the topic of this survey?”; and (3) semistructured interviews. The surveys gathered quantitative data to ensure consistent comparability across respondents and to offer a broad understanding of the research problem. The open-ended question provided qualitative insights that closed-ended questions might not have captured and helped inform the interview guide. Finally, we conducted interviews not only to gain deeper and contextualized insights into the research topic but also to identify approaches for addressing the issues and to inform actionable recommendations for multiple stakeholders. Quantitative and qualitative components were integrated through sequential exploration, triangulation, complementarity, and the development of practical implications to enhance the validity and depth of interpretation.

### Survey

In 2021, we conducted an anonymous web-based survey with physicians from various disciplines across Germany. A total of 11 volunteers pretested the survey for usability and comprehensibility using the think-aloud method [25]. Developed and administered with SoSci Survey (SoSci Survey GmbH), the survey was distributed using convenience sampling [26]. The German Radiological Society, the Center for Integrated Oncology Aachen, Bonn, Cologne, Düsseldorf, and the German Society of Radiation Oncology disseminated it via websites, newsletters, and mailing lists. Reminders were sent, and additional internists, anesthesiologists, and pathologists were contacted through institutional mailing lists. Data collection spanned 11 months, from February 2021 to January 2022.

To our knowledge, no validated questionnaire currently exists for the purpose of this study. Therefore, we developed a survey based on the CHERRIES (Checklist for Reporting Results of Internet E-Surveys) guidelines [27] (supplementary materials 1 and 2 in Multimedia Appendix 1). The survey was designed with input from a multidisciplinary team of physicians, AI researchers, ethicists, and a health scientist and was informed by a thorough literature review on physicians’ attitudes toward technology and AI in health care [20,28-32]. In the survey, AI was primarily presented in the context of diagnostics. This included pattern recognition tools for image-based analysis (eg, x-ray imaging, magnetic resonance imaging, and computed tomography scans) and systems that integrate multimodal diagnostic data (eg, genome data and laboratory parameters) to support screening and clinical decision-making. Attitudes were assessed using 5-point Likert scales [33]. These captured both affective responses to AI (ie, emotional valence and intensity) and cognitive perceptions of the potential capabilities and limitations of AI (eg, perceived loss of control or enhancement of medical specialties). Beyond attitudes, the survey included items on demographic and professional characteristics, perceived benefits and drawbacks of AI, perceived distributions of responsibility, the influence of AI on physician-patient communication, and the role of AI in medical education using single- and multiple-response questions. The survey also included conditional questions (eg, “Please state an example of AI-supported products that you use privately”) and nonresponse options. Some questions allowed respondents to elaborate within an “other” category. The respondents were not required to answer every question and were able to review their responses before submission. The survey concluded with an optional open-ended question.

SoSci Survey recorded 1492 link visitors, with 659 agreeing to participate. During data cleaning, we excluded incomplete records (eg, those not reaching the last page), records missing critical sociodemographic information, and records from nonphysicians and medical students. No survey was completed in an unusually short or long time, as determined by an index of relative completion speed [34]. This resulted in a final dataset of 498 cases and a completion rate of 76%, which was calculated by dividing the number of completed surveys by the number of respondents who agreed to participate.

Respondents’ characteristics were summarized using descriptive statistics, that is, proportions (%) and means (SDs). Multiple-response questions were reported as the percentage of total answers (% of answers) and the percentage of physicians selecting these answers (% of physicians). Negatively worded items on the 5-point Likert scale were reverse coded to ensure that higher values indicated stronger agreement. An exploratory factor analysis (EFA; a principal component analysis with varimax rotation) was used to assess the extent to which the items measured constructs related to physicians’ attitudes toward AI and to identify underlying latent variables. The suitability for EFA was evaluated based on a Kaiser-Meyer-Olkin value >0.5 and a *P* value from the Bartlett test of sphericity <.05. The factors were extracted based on eigenvalues >1 and the scree plot. The items with communalities and factor-loading scores >0.4 were retained. The internal consistency of each factor was assessed using Cronbach α. For further analyses, the missing values (“Don’t know” responses <0.5%) were imputed using the overall mean of each scale. The differences between the demographic and professional groups or subgroups and the extracted factors were analyzed using the Kruskal-Wallis test or the Mann-Whitney *U* test for pairwise comparisons. The pairwise comparisons were adjusted using the Bonferroni correction and performed on subgroups with >20 participants. The effect sizes were calculated for the statistically significant pairwise comparisons. Two-sided *P* values <.05 were considered statistically significant [35]. The analyses were performed using IBM SPSS Statistics (version 29).

### Open-Ended Survey Question

This question contextualized the survey results and guided the interview design. Of the 498 survey participants, 93 (18.7%) provided comments, a response rate consistent with a previous study on open-ended survey item nonresponse [36]. A total of 15 comments that were brief or irrelevant, such as expressions of interest in the study results or well-wishes for success, were excluded based on an independent evaluation to ensure a focus on meaningful, thematically relevant responses. Using a data-driven, inductive coding approach [37], the recurring themes were identified and categorized by reading the data sentence by sentence.

### Qualitative Interviews

In December 2023, HH conducted qualitative, semistructured, and problem-centered interviews with physicians, distinct from those who participated in the survey. A maximum variation sampling strategy [38] was used to ensure participant diversity by age, years of experience, and medical discipline, capturing a broad range of perspectives on AI in medicine. Participants were purposively selected [26] and contacted via email and personal networks, with 13 physicians agreeing to participate.

We developed an interview guide (supplementary material 3 in Multimedia Appendix 1) informed by the survey results and themes identified from the open-ended survey responses to gain a holistic understanding of the impact of AI on physicians. It served as a key instrument for structuring in-depth interviews, ensuring comparability across interviews while allowing flexibility to explore emerging themes and to elaborate on interviewees’ responses in greater depth. The focus areas included motivation and restraint, needs and challenges, human-machine interaction, transparency and explainability, and preparedness for upcoming changes. Each interview was scheduled for 30 minutes and conducted via Zoom (Zoom Video Communications, Inc). Audio recordings, made with participants’ permission, were transcribed verbatim using Happy Scribe (Happy Scribe Ltd), following transcription rules [39]. The transcripts were subsequently reviewed to ensure their correctness.

A thematic analysis was conducted on the interview transcripts using MAXQDA 24 (VERBI Software GmbH; version 24.8.0). We familiarized ourselves with the data by examining it sentence by sentence and inductively coded it through in-depth textual analysis. Descriptive codes were assigned to text segments to capture key themes and patterns, forming the basis of a category scheme [37]. The data extracts and coded segments were re-evaluated iteratively throughout the process, and the collated codes were organized into salient patterns of meaning. Amid iterative control loops, a deductive-inductive interplay between the interview data and open-ended responses provided a comprehensive overview of physicians’ perspectives and lived experiences. Initial ideas were reorganized, rejected, or subsumed into new themes as the analysis progressed, and the themes were ultimately refined and defined for clear reporting. Coding reliability was ensured through multiple iterative rounds of independent coding by HH, followed by analytical discussions between HH and AK and between HH and FK to resolve discrepancies and achieve consensus on the final themes. To account for researcher reflexivity, the analysis involved continuous self-reflection. HH maintained analytic memos to document ideas, assumptions, and coding decisions. This collaborative process led to challenging interpretations, reflecting on positionality, and mitigating individual bias. Finally, we concluded the presentation of each category with a propositional statement that synthesized key insights emerging from the interviews [38]. These statements highlight physicians’ perspectives on factors that facilitate or hinder the adoption of AI. Each proposition was completed with a recommendation that offers potential starting points for practical implementation or policy development, based on participants’ insights. The study followed the Standards for Reporting Qualitative Research checklist [40] for rigorous qualitative reporting (supplementary material 4 in Multimedia Appendix 1).

### Ethical Considerations

This study obtained ethics approval from the Ethics Committee of the University Hospital RWTH Aachen (CTC-A no. 20-393) and was conducted in accordance with the tenets of the Declaration of Helsinki and its subsequent revisions and the General Data Protection Regulation. The first page of the survey provided information on its purpose, estimated duration, anonymity, voluntary nature, and data management and requested informed consent. Before the interviews, the participants received detailed study information; privacy and confidentiality were ensured at all times, and written informed consent was obtained. Each interview participant was offered an incentive of €50 (US $55) as compensation for their time; this was disclosed upfront and was unrelated to their responses [38].

## Results

### Survey

Of the 498 survey respondents, 161 (32.3%) were women and 334 (67.1%) were men. The majority were aged 30 to 39 years (126/498, 25.3%), 40 to 49 years (129/498, 25.9%), or 50 to 59 years (146/498, 29.3%). The physicians represented all clinical hierarchical levels, with most working in radiology (303/498, 60.8%), followed by radiotherapy and nuclear medicine (56/498, 11.2%), surgical specialties (33/498, 6.6%), internal medicine (33/498, 6.6%), and anesthesiology (31/498, 6.2%; see Table S1 in Multimedia Appendix 1 for the categorization of medical disciplines). AI was used for medical purposes by 32.1% (160/498) of the respondents. In contrast, 67.3% (335/498) of the respondents had never used AI professionally, although 30.7% (153/498) intended to do so. Notably, a large majority (471/498, 94.6%) expressed interest in AI. In addition, 43% (214/498) of the respondents reported being quite or very familiar with AI, while 7% (35/498) stated that they were unfamiliar with it (Table 1).

The majority of respondents considered AI to be an aid for medical staff (447/498, 89.8%) and a tool for supporting preventive diagnostics (406/498, 81.5%), with only a small proportion of respondents (29/498, 5.8%) viewing it as a sole decision maker. The physicians acknowledged the advantages of AI, including faster processing of routine tasks (414/498, 83.1%), increased safety in diagnostic and therapy (373/498, 74.9%), and support in treatment decisions (330/498, 66.3%). Key concerns included liability issues in the event of errors (411/498, 82.5%) and a lack of transparency in AI decision-making (377/498, 75.7%). Many believed that AI would not change physician-patient communication (263/498, 52.8%) or trustworthiness (280/498, 56.2%), whereas 58.6% (292/498) of the respondents expected it to improve communication efficiency. A majority believed physicians should be held responsible for incorrect diagnoses (312/498, 62.7%) and treatment decisions (329/498, 66.1%) involving AI, followed by medical device manufacturers (diagnosis: 285/498, 57.2%; treatment: 261/498, 52.4%) and hospitals (diagnosis: 183/498, 36.7%; treatment: 195/498, 39.2%). Few respondents attributed responsibility to health insurance companies or patients. Although 57.8% (288/498) of the respondents reported that AI was insufficiently integrated into medical education, most anticipated a major shift in clinical practice within 5 years (111/498, 22.3%) or in 5 to 10 years (252/498, 50.6%). Tables S2-S5 in Multimedia Appendix 1 provide a detailed breakdown of the responses.

The EFA identified underlying constructs among the 13 attitudinal items, with descriptive response distributions provided in Table 2. One item (“There are sufficient legal and technical-organizational guidelines for the use of AI in medicine”) loaded solely onto a third factor and was removed. The final model (comprising 12 items) met all necessary criteria. The Kaiser-Meyer-Olkin value (0.88) confirmed sampling adequacy, and Bartlett test of sphericity (χ^²^_66_=2189.6; *P*<.001) indicated sufficient item correlation for the EFA. The results revealed 2 factors with eigenvalues >1, as did the scree plot, explaining 54.71% of the total variance. All items loaded well above the critical value of 0.4, with loadings ranging from 0.545 to 0.796. Two items (“open” and “skeptical”) showed cross-loadings but were retained because of contextual relevance. All negatively worded items loaded onto factor 2. Factor 1, labeled *AI enthusiasm and acceptance*, comprises 7 items reflecting physicians’ openness, curiosity, and perceived benefits (eg, AI effectiveness and AI’s supporting role in medical decision-making). Factor 2, labeled *AI skepticism and apprehension*, includes 5 items related to uncertainty, skepticism, and perceived risks (eg, the loss of control and threat to medical specialties). Cronbach α indicated high reliability for both factors (α=0.83 for factor 1 and α=0.77 for factor 2). A Wilcoxon signed rank test showed a significant difference between the 2 factors (*z*=8.849; *P*<.001; *r*=0.397), indicating a medium effect size. The physicians strongly agreed with factor 1, with a median of 4 (IQR 3.57-4.29), indicating high enthusiasm and acceptance of AI. Scores for factor 2 were also above the midpoint of the scale, with a median of 3.62 (IQR 3.20-4.20), suggesting that although some apprehension exists, the physicians were not strongly skeptical overall. Higher scores on both factors indicate a favorable attitude toward AI (Table 3).

AI enthusiasm and acceptance was significantly influenced by gender, (AI-related) research, personal or professional AI use, and familiarity with AI. Positive attitudes were significantly higher among men (median 4) than among women (median 3.75; *U*=23,932.5; *z*=−1.99; *P*=.047; *r*=0.09), indicating a small effect size. Physicians engaged in clinical or experimental research had significantly more positive attitudes (median 4) than those not engaged in research (median 3.62; *U*=25,502.5; *z*=−2.956; *P*=.003; *r*=0.21), indicating a small to medium effect size. Physicians involved in AI research showed significantly greater enthusiasm (mean rank 54.32-111.52; *P*<.001; *r*=0.39) than those not engaged in AI research, indicating a medium effect size. Physicians who actively used AI in professional practice exhibited significantly higher enthusiasm (mean rank: 196.17-253.88; *P*=.001; *r*=0.20), indicating a small to medium effect, compared to those who did not. Those who planned to use AI in the future demonstrated even higher enthusiasm (mean rank 196.17-303.50; *P*<.001; *r*=0.37), indicating a medium effect size. Familiarity with AI was among the strongest predictors of positive attitudes. Physicians with high AI familiarity showed significantly greater enthusiasm and acceptance (mean rank 169.86-323.55; *P*>.001; *r*=0.54), demonstrating the largest effect.

Physicians’ skepticism and apprehension about AI were similarly shaped by their level of AI engagement. The willingness to use AI professionally correlated with lower skepticism (mean rank 218.86-275.93; *P*=.001; *r*=0.37), and greater familiarity with AI significantly reduced concerns (mean rank 186.23-296.90; *P*=.008; *r*=0.39), with both representing medium effect sizes. Chief physicians were less skeptical than junior physicians (mean rank: 210.60-277.32; *P*=.01; *r*=0.23), reflecting a small to medium effect. No differences in skepticism were found between men and women (men: median 3.50, women: median 3.75; *U*=24.800; z=−1.40; *P*=.16). Age, years of clinical experience, and medical discipline also showed no notable associations. An overview of all group and subgroup comparisons is provided in Tables S6 and S7 in Multimedia Appendix 1.

**Table 1:** Demographic and professional characteristics of the respondents (N=498).

Characteristic		Respondents, n (%)
**Gender**		
	Women	161 (32.3)
	Men	334 (67.1)
	Nonbinary	3 (0.6)
**Age (years)**		
	<30-39	143 (28.7)
	40-49	129 (25.9)
	50-59	146 (29.3)
	>60	80 (16.1)
**Medical disciplines^a^**		
	Radiology	303 (60.8)
	Radiotherapy and nuclear medicine	56 (11.2)
	Anesthesiology	31 (6.2)
	Surgical disciplines	33 (6.6)
	Internal medicine	33 (6.6)
	Other	42 (8.4)
**Physician’s position**		
	Residents^b^	72 (14.5)
	Attendings^c^ (specialist)	166 (33.3)
	Attendings^c^ (senior)	155 (31.1)
	Attendings^c^ (chief)	105 (21.1)
**Professional experience (years)**		
	<10	123 (24.7)
	10-20	145 (29.1)
	>20	230 (46.2)
**Working in research**		
	Yes, in clinical research, experimental research, or both	197 (39.6)
	No	301 (60.4)
**Research related to AI^d,e^**		
	Yes	101 (51.3)
	No, but I can imagine doing so in the future	74 (37.6)
	No	22 (11.2)
**Interest in the topic of** **AI** **in medicine**		
	Yes	471 (94.6)
	No	9 (1.8)
**Use of** **AI** **in everyday life**		
	Yes	392 (78.7)
	No, but I plan to do so in the future	32 (6.4)
	No	73 (14.7)
**Use of** **AI** **in a professional context**		
	Yes	160 (32.1)
	No, but I plan to do so in the future	153 (30.7)
	No	182 (36.5)
**How familiar are you with the use of** **AI** **in medicine?^f,g^**		
	Not at all familiar	35 (7)
	Somewhat familiar	146 (29.3)
	Moderately familiar	98 (19.7)
	Quite familiar	175 (35.1)
	Very familiar	39 (7.8)

^a^A complete list of medical disciplines within these categories is provided in Table S1 in Multimedia Appendix 1, including additional details on the “other” category.

^b^Residents are physicians undergoing specialized training. Most residents indicated their medical discipline; those who did not were classified as “other.”

^c^An attending physician is a trained physician practicing in their specialty.

^d^AI: artificial intelligence.

^e^Percentages reflect only the physicians engaged in research (197/498, 39.6%). The corresponding percentages relative to the total population are: “Yes” (20.3%); “No, but I can imagine it for the future” (14.9%); and “No” (4.4%).

^f^“Don’t know” responses, as a nonresponse option, were excluded for a better overview, as they accounted for ≤4% of the total response distribution.

^g^Missing values are not shown, as they account for ≤0.5% of the total response distribution.

**Table 2:** Physicians’ attitudes toward AI^a^ in medicine (N=498).

			Values, mean (SD; 95% CI)^b^		Values, n^c^
**Emotions regarding digital changes (including** **AI** **)**					
	Anxious^d^	4.03 (0.984; 3.94-4.12)		497	
	Tense^d^	3.86 (1.021; 3.77-3.95)		498	
	Skeptical^d^	3.36 (1.132; 3.26-3.46)		498	
	Open	4.20 (0.755; 4.13-4.27)		498	
	Hopeful	3.84 (0.893; 3.76-3.92)		498	
	Curious	4.38 (0.778; 4.31-4.44)		498	
**Perceptions of** **AI** **in medicine**					
	AI can effectively support physicians.	4.37 (0.667; 4.31-4.43)		494	
	The use of AI in medicine is superior to physicians clinical experience.	2.47 (0.903; 2.39-2.55)		481	
	I would seek a second opinion from an AI in decision-making processes.	3.73 (1.020; 3.64-3.82)		494	
	The use of AI leads to a loss of control in my job.^d^	3.67 (0.995; 3.59-3.76)		490	
	Some medical specialties will be threatened by new technologies such as AI in the future.^d^	3.14 (1.186; 3.03-3.24)		485	
	Some medical specialties will be enhanced by new technologies such as AI in the future.	4.38 (0.645; 4.32-4.43)		496	
	There are sufficient legal and technical-organizational guidelines for the use of AI in medicine.	2.06 (1.008; 1.96-2.15)		435	

^a^AI: artificial intelligence.

^b^Responses were given on a 5-point Likert scale (1=strongly disagree and 5=strongly agree), with 3 representing the midpoint of the scale.

^c^Only valid responses (n) were used to calculate the descriptive statistics. Missing values or “don’t know” responses were excluded from the calculation of the mean (SD) and 95% CI.

^d^All reversed items were recoded before the statistical analyses to ensure consistent interpretation. Higher values consistently indicate a more positive attitude toward AI.

**Table 3:** Summary of the exploratory factor analysis results on physicians’ attitudes toward AI^a^ in medicine.

Item^b^		Rotated factor loadings^c^	
		Factor 1	Factor 2
Hopeful		*0.765* ^d^	0.291
I would seek a second opinion from an AI in decision-making processes.		*0.711*	0.163
AI can effectively support physicians.		*0.707*	0.031
Some medical specialties will be enhanced by new technologies such as AI in the future.		*0.680*	0.235
Curious		*0.654*	0.400
The use of AI in medicine is superior to physicians clinical experience.		*0.603*	−0.123
Open		*0.582*	0.519
Tense^e^		0.153	*0.796*
Anxious^e^		0.175	*0.770*
The use of AI leads to a loss of control in my job.^e^		0.243	*0.688*
Some medical specialties will be threatened by new technologies such as AI in the future.^e^		−0.100	*0.651*
Skeptical^e^		0.479	*0.545*
**Summary statistics^f^**			
	Eigenvalues	4.931	1.634
	Variance (%)^g^	41.091	13.621
	Cronbach α	0.828	0.769

^a^AI: artificial intelligence.

^b^An exploratory factor analysis was conducted on 12 of the original 13 items using orthogonal rotation (varimax).

^c^The values represent the factor loadings after rotation and the distribution of the items between the 2 components. Missing values were excluded pairwise.

^d^Italicized values indicate the factor onto which each item was loaded after rotation.

^e^Reversely coded items were recoded before conducting the factor analysis.

^f^Summary statistics correspond to each factor (column).

^g^A total of 2 components had eigenvalues above the Kaiser criterion of 1 and jointly explained 54.71% of the total variance.

### Findings From Open-Ended Responses and Interviews

#### Overview

The 13 interviews averaged 31 (SD 5.46) minutes and generated 178 pages of 1.5-spaced text. The physicians were aged 28 to 58 years (mean 39, SD 7.38 years), and the majority were men (9/13, 69%). The respondents varied in experience (mean 11.92, SD 7.41; range 3-30 years), medical disciplines, and work settings (Table 4).

Qualitative analysis identified 6 themes that reflect physicians’ perspectives on the impact of AI on their work. These themes, summarized subsequently with selected physicians’ quotes, include (1) physicians’ reflections on the status quo, (2) AI dependency and negligence, (3) role changes and needs, (4) AI transparency and decision-making, (5) the physician-patient relationship, and (6) a framework for responsible AI integration. These themes informed the development of definitional propositions and practical recommendations, offering actionable insights for policy makers. The coding scheme, including subcategories, category descriptions, and additional sample quotes, is provided in Table S8 in Multimedia Appendix 1.

**Table 4:** Descriptive characteristics of the interview participants (N=13).

Number	Gender	Age (years)^a^	Years in practice^b^	Work setting	Medical discipline	Physician position	Duration (min)^c^
1	Man	38	9	University clinic^d^	Pediatrics	Attending^e^	32
2	Man	41	11	University clinic^d^	Radiology	Attending	28
3	Woman	41	14	University clinic^d^	Pathology	Attending	39
4	Woman	44	18	Private practice	Internal medicine and diabetology	Attending	27
5	Man	58	30	Hospital	Internal medicine and diabetology	Attending	19
6	Man	40	14	University clinic^d^	Otorhinolaryngology	Attending	26
7	Man	33	6	University clinic	Urology	Attending	34
8	Man	39	13	University clinic^d^	Radiology	Attending	40
9	Man	36	7	Hospital	Anesthesiology and intensive care	Attending	32
10	Woman	28	4	University clinic^d^	Internal medicine and hepatology	Resident^f^	33
11	Man	46	19	Private practice^d^	Radiology and nuclear medicine	Attending	37
12	Man	36	7	Hospital	Anesthesiology and intensive care	Attending	29
13	Woman	33	3	Hospital	Radiology	Resident	35

^a^The mean age was 39.46 (SD 7.38) years.

^b^The mean time working in practice was 11.92 (SD 7.41) years.

^c^The mean interview duration was 31.39 (SD 5.46) minutes, excluding the introductory part of the interview.

^d^Physicians who work in research and whose research is related to artificial intelligence.

^e^An attending physician is a trained physician practicing in their specialty.

^f^A resident is a physician undergoing specialized training.

#### Physicians’ Reflections on the Status Quo

The first category captures physicians’ perceptions of Germany’s health care system and focuses on the current state of digital transformation, AI integration, and the structural challenges hindering its adoption in clinical practice. Interview data indicate that the German transformation is lagging in international comparisons because of the slow pace, poor digital infrastructure, and AI being barely integrated into routine practice, dampening the physicians’ enthusiasm:

Germany and Europe have simply slept through many trends and developments in digitalization over the past two decades. Unfortunately, this will likely repeat with AI.

SR (survey respondent) 91

The category also reflects the physicians’ know-how and readiness for AI. Transforming the health care system presents opportunities to manage medicine’s growing complexity, simplifying administrative tasks and allowing more time for patient interaction:

AI could reduce my workload, allowing me to spend more time with patients instead of just five to seven minutes.

IP (interview partner) 1

The physicians also highlighted the potential of AI for enhancing diagnostic accuracy:

Standardized AI that reviews all findings could prevent clinically relevant incidental findings from being overlooked.

IP2

The physicians viewed AI as an inevitable part of their future:

It’s hard to imagine a radiologist working alone in ten years without AI assistance.

IP13

However, many respondents expressed frustration with the inefficiency of current systems, which lack interoperability with existing hospital and clinical IT systems:

Digitalization is still so poor [...], there is no way to transfer images from hospital A to hospital B. [It] requires manual downloads, uploads, or even sending CDs.

IP8

Fragmented systems necessitate duplicative documentation:

We have to document everything two or three times in all kinds of computer programs because they are incompatible.

IP12

In addition, bureaucratic requirements add to physicians’ workload rather than streamlining tasks, leading to overregulation and stifled innovation:

Digitalization [is] frustrating [...] it’s more of a hindrance than it makes work efficient [and adds] at least three or four minutes more work that wasn’t there before.

IP9

Although AI is often expected to free up time for patient interaction, some physicians argue that it primarily increases workload expectations instead:

It’s always said AI [...] will give us more time to engage with patients. But in reality, the workload hasn’t decreased. Instead of reviewing 50 CTs, we’ll review 60, leaving no more time for meaningful interaction. [IP8]

Ultimately, many participants spoke about digitalization or proposed AI applications but noted that the actual integration of AI into their daily work is limited, mainly confined to research settings or specific medical disciplines such as radiology.

Costs also hinder progress. Physicians stated that smaller hospitals are particularly vulnerable, as they lack the subsidies available to larger research-focused institutions, creating disparities in the accessibility of AI. Additionally, the lack of an immediate return on investment makes AI investments difficult to justify:

The healthcare budget is large but limited. This means that every additional expense, even if it serves quality, is hard to justify [...] unless it offers immediate value, which is often difficult to quantify.

IP8

Furthermore, large AI models require massive financial investments, limiting development to a small number of major industry players:

Only those who can afford billions in training-infrastructure [...] can build these models, leaving academic institutions behind.

IP2

Some participants fear the growing influence of commercial interests in health care optimization and the concentration of power among large technology companies, potentially sidelining smaller health care providers and academia:

The only ones who can expect anything from AI are the software companies that are entering the healthcare market and siphoning off another share of the limited resources.

SR670

- Proposition 1: financial and structural investments are necessary to allow health care professionals to integrate AI into the existing clinical workflows.
- Recommendation 1: explore ways to support the integration of AI into routine clinical practice by improving workflow alignment and interoperability.

#### AI Dependency and Negligence

A recurring theme was the concern about physicians’ dependence on AI and the associated risk of skill loss, particularly among the younger medical generation. Some perceived it as valuable for reducing errors, concentration difficulties, and stress because of its constant availability:

AI will do some of the work of the employee, and the device cannot get sick, but it will always be there and does its job well. If it works properly and is used properly.

IP6

However, others warned that the overuse of AI could erode critical diagnostic skills by fostering overdependence on AI outputs. They worried this might encourage negligence and compromise the quality of care, especially if the pressure for productivity leads to blind trust in AI-based solutions:

I simply wouldn’t check it anymore because I would just blindly rely on it. [...] Just because so much is calculated and processed in the background and then the output, is blindly accepted.

IP9

If you wanted to treat everyone the way you wanted your relatives to be treated, I don’t think you could do it.

IP8

Participants pointed to an existing quality control measure that could prevent overreliance on AI and AI-generated errors from going unnoticed:

Relying 100% on AI would be problematic. We have a two-step system where an assistant and a senior physician both review the findings to catch any small errors.

IP10

Furthermore, the physicians mentioned a fear of uncritical acceptance of AI diagnosis, while others reject algorithmic advice without considering it:

Physicians are very easy to convince of a certain statement, even if it’s wrong because they think and know that it came from an AI. Some people have automation bias [...], who are more likely to follow the AI, and others have automation aversion, then even if it is correct, they say the opposite just to be against the AI.

IP8

Another factor contributing to increased negligence of physicians is the phenomenon of satisfaction of search. Once an AI detects an abnormality, physicians may fail to continue searching for additional abnormalities after identifying an initial one. AI can help identify a clear starting point for physicians, but this may, in turn, reduce their tendency to analyze problems thoroughly:

Satisfaction of search. If you’ve found a conspicuous feature [...], you might tend to overlook other things because you already have the feeling,[...] we’ve already found something and that more or less solves the case.

IP13

- Proposition 2: the adoption of AI in medical practice is perceived as potentially leading to overdependence on algorithmic advice, highlighting the need for robust guardrails to preserve critical diagnostic skills and accountability.
- Recommendation 2: establish clinical safeguards and awareness strategies to address concerns around overreliance and automation bias.

#### Role Changes and Needs

This category reflects on the physicians’ perceived roles in future work with AI, anticipated changes, and factors essential to maintaining their role. Amid workforce changes, the physicians want to preserve human experience and final decision-making. Medical experience is seen as irreplaceable, as the physicians are familiar with patients’ medical family histories, and the collegial and interdisciplinary exchange is of great value when discovering new findings:

Experience is different from AI. AI can only do what it’s programmed to do. [...] Meanwhile, after 30 years as a physician, I bring experiential knowledge to the table—things that aren’t in any textbook but are evident from experience.

IP5

This quote reflects a perspective where experiential knowledge is valued over the perceived capabilities of AI. Another physician recognized the potential of machine learning, particularly in language models, to transform unstructured data, such as physicians’ notes or radiology reports, into structured formats, without the need for oversight. At the same time, AI is actively reshaping workplace structures and job roles, influencing how physicians and health care staff allocate their time and expertise. Some physicians see AI as a tool for optimizing workforce distribution, allowing human resources to reallocate toward higher-value and patient-centered tasks, while AI handles higher-volume and repetitive tasks:

I’ve followed a lot of discussions about AI [...] initial excitement, [...] followed by fears of job loss. But I never believed in the latter. Many jobs have disappeared over time, and that’s just how things evolve. It’s not necessarily a bad thing.

IP8

Another physician in private practice highlighted how AI enabled workforce restructuring:

We [...] reassigned six employees without letting them go. AI allowed us to free up their roles in patient registration. This isn’t about replacement but reallocation. And that’s where I actually see AI’s greatest value. As a physician, I’m not worried about being replaced by AI. On the contrary, I see it as an opportunity to enhance diagnostic quality.

IP11

Respondents criticized the lack of awareness of AI tools and the possibilities available. They emphasized that as primary users, they expect to be actively informed and involved in discussions about AI implementation to ensure its practical relevance in their work context:

The key stakeholders must be those who will actually use it, namely physicians, but also nursing staff. If it’s meant to be applied in this context, they need to be addressed.

IP1

Many emphasized the need to enhance their skills to facilitate effective AI adoption through extensive training and support. Physicians criticized the shortcomings of current implementation practices, often relying on minimal instruction. Often, AI is not even a topic of interest at their work, leaving them unprepared:

Training is crucial to understand what we are actually doing with AI.

IP9

It’s not enough to have a single lecture. There needs to be hands-on support, someone accompanying us in daily practice, showing how to use it effectively, and explaining its applications. Without this, the tools are underutilized.

IP9

The lack of AI in current medical training was also criticized:

AI is barely touched upon in medical school. We only had one lecture on biostatistics where AI was mentioned in passing.

IP10

- Proposition 3: the physicians recognize evolving professional roles driven by AI adoption and, thus, require targeted education on effectively integrating AI into clinical practice.
- Recommendation 3: integrate AI literacy into undergraduate and continuing medical education to prepare physicians for evolving clinical roles and to address knowledge barriers identified in this study.

#### AI Transparency and Decision-Making

This category accentuates the complexity and fallibility of both AI and human decision-making. It highlights the ways in which physicians critically reflect on decision-making processes in the context of AI because of changing circumstances. The interviewees expressed varied opinions on the importance of trust and transparency in AI-based decisions. Some emphasized the need for transparent systems to foster trust but acknowledged the difficulty of fully understanding their complexity. The interviewees preferred understanding AI reasoning when it deviated from their assessments and insisted on remaining actively involved in the decision-making process to allow critical evaluation, falsification, and verification:

If the AI says “this cannot be,” I would consider whether the AI is correct, and if it isn’t, then I would have to figure out how to disprove it.

IP5

Well, one would have to be able to fully understand it. If that is the case, then I would ultimately trust this AI. But [...] if I just get diagnosis XY and I have a completely different one, then of course I would be skeptical at first.

IP6

Other physicians further pointed out the inherent complexity of both AI and human decision-making, emphasizing their shared lack of transparency:

Many things, we believe we do correctly as physicians, are perhaps flawed. [...] But if we can receive support in that regard, why not?

IP7

At the same time, the physician acknowledged that there is rarely a single golden path in medicine:

If you ask five physicians, you get five answers, and each approach is probably acceptable in its own way.

IP7

While transparency is a cornerstone of trust for many physicians, others prioritize performance over understanding the underlying mechanisms, accepting opacity:

I don’t think it’s that important. There’s so much in medicine we can’t understand. I don’t understand how an MRI works, yet I trust that the image is what it is. [...] This [...] topic is always heavily debated, but honestly, I consider it futile. [...] As long as the AI is always correct, congratulations, I don’t need to understand it.

IP8

While perspectives on AI transparency vary, the physicians generally agree on its role as a valuable support in decision-making. The collaborative potential of AI was compared with consulting an experienced colleague, providing assurance as a “supporting eye” (IP5), and increasing diagnostic confidence while maintaining human oversight:

If I see the computer reaches the same conclusion as I do, that’s great. Or if it identifies things I’ve missed, even better.

IP11

It’s really a way to secure your work, a sort of [...] control measure.

IP13

- Proposition 4: the physicians stress that the underlying reasoning of AI must be sufficiently transparent to enable them to interpret and integrate AI-generated assessments effectively.
- Recommendation 4: encourage the development of AI systems that provide clinically relevant and interpretable insights and ensure physician oversight to foster trust and accountability in decision-making.

#### The Physician-Patient Relationship

Integrating AI into medicine presents opportunities and challenges in human-machine interaction, the physician-patient relationship, and interdisciplinary collaboration. Opportunities include improved communication and increased patient trust. However, patients’ greater access to AI-generated medical information online (eg, ChatGPT) is a challenge, as it could increase misunderstanding and misinformation, especially in the case of low AI literacy, potentially eroding trust in the physician-patient relationship:

I think it will lead to a greater need for explanation because patients are becoming increasingly aware that AI algorithms are being used. This will take up additional time.

IP2

The problem [...] arises when patients also gain access to AI. If they become biased by it, the physician-patient relationship could be disrupted.

IP5

Similarly, the physicians noted that well-trained AI programs can positively influence interactions by reinforcing the physician’s diagnosis. When the AI and the physician’s judgment align, it reduces the effort needed to persuade patients of the accuracy of the diagnosis and fosters better consultations.

While AI can streamline workflows, concerns were raised about its effect on human interaction, especially with older patients during consultation. Some were also skeptical that AI would free up time for patient interaction, citing systemic challenges, such as the lack of incentives for meaningful engagement:

Because whenever there is a way to save time, you can be sure it will be used, and contact with patients will definitely be reduced.

IP12

We have a digital pre-screening algorithm [...] so they can [...] say where they have the complaints, what does it look like, have they had previous surgery? Older people can’t do that at all. And our clientele consists mainly of older and not younger people.

IP11

- Proposition 5: as AI is expected to influence the patient-physician interaction, in both positive and challenging ways, its use by both parties should be transparently integrated into clinical consultations. To preserve trust and avoid misunderstandings, AI must be understood by both patients and physicians as a complementary tool.
- Recommendation 5: promote clear communication with patients when using AI-based technologies for decision-making and the recommendation of therapy.

#### Framework for Responsible AI Integration

The physicians support the adoption of AI but require a clear regulatory framework that covers data protection, liability, transparency, and seamless workflow integration to uphold professional responsibility and patient trust. The physicians call for explicit information on legal liability when AI-based tools are deployed:

It must be clearly regulated that the physician has the final say, and AI is merely seen as a medium that contributes an opinion. [...] How does it apply to AI—does it remain purely advisory, or does it carry some decision-making power and how is it weighted?

IP12

This aligns with broader perspectives among physicians that accentuate their ultimate authority in final decisions:

The responsibility lies with the physician or another human. AI can provide diagnoses and support, but I must always verify its conclusions.

IP4

Final therapy decisions will always lie with the patient and physician. [...] AI is a tool, not a decision maker.

IP6

While they acknowledged the role of AI in supporting clinical decisions, they stressed that legal frameworks must explicitly define the extent of physicians’ liability. One physician proposed a shared liability model between AI developers and health care providers:

Perhaps the company implementing the AI system [...] should share responsibility for its outcomes. If I include AI-driven findings in my report, [...] the company backing the technology should also be held accountable.

IP9

The physicians also highlighted the importance of ensuring high-quality, reliable AI systems trained on representative and unbiased data to prevent perpetuating inequalities in care:

We need to be clear about the gold standard—how a diagnosis is ultimately confirmed and the criteria used to determine its valid.

IP1

We need to pay close attention to biases, like those that disadvantage African Americans as seen in the U.S. These biases can carry over into AI algorithms and must be addressed during development.

IP10

New technologies must integrate into and complement existing workflows, ensuring user-friendliness, as current systems often fall short. Therefore, the physicians emphasized that AI must be practically embedded into routine clinical procedures to ensure effectiveness:

AI integration must align seamlessly with clinical workflows to avoid disruptions and maximize its potential.

IP2

AI-based systems, like in cardiac diagnostics, are great and adopted immediately if they work well. [...] Software vendors must ensure these to be seamlessly integrate into our clinical reporting workflows.

IP11

- Proposition 6: the physicians require clear and actionable guidelines on liability, bias, and data protection to facilitate the adoption of AI.
- Recommendation 6: clarify legal accountability and consider policy incentives and funding to support the responsible adoption of AI tools in existing health care infrastructures.

## Discussion

### Overview

As AI proliferates in medical practice, it offers enhanced diagnostics, personalized care, and the potential to transform overall health care delivery. Despite its potential, pressing ethical and practical implications remain, and a comprehensive understanding of the attitudes of physicians toward AI is lacking. Our survey results showed that increased personal and professional engagement with AI was associated with higher AI enthusiasm and acceptance and reduced AI skepticism and apprehension. The interviews revealed six themes that shaped the attitudes of physicians toward AI: (1) reflections on the status quo, (2) AI dependency and negligence, (3) role changes and needs, (4) AI transparency and decision-making, (5) the physician-patient relationship, and (6) a framework for responsible AI integration. By using an explanatory mixed methods design that integrated both quantitative and qualitative data, we enhanced the validity, interpretability, and triangulation of our findings and derived actionable propositions and recommendations [24]. Accordingly, we jointly synthesized and discussed the quantitative and qualitative findings.

### Principal Findings

This study highlighted a generally positive attitude toward AI, with nearly all physicians expressing interest in the topic, consistent with recent literature [32,41-43]. Familiarity with AI significantly influenced positive attitudes, as those who actively used AI reported greater enthusiasm and less skepticism toward its integration into medical practice. A lack of familiarity and knowledge contributed to AI skepticism and apprehension [30,44], while a positive attitude toward an innovation is one of the well-documented factors determining its adoption [29]. The interviews provided deeper insights into the perceptions and expectations of the physicians. While most physicians held a favorable view of AI, some expressed skepticism and apprehension, possibly because of limited routine use of AI in medical practice or because past experiences with AI were unsuccessful. However, skepticism should not be viewed as merely an adverse reaction to new technologies. Instead, it fosters critical engagement, encouraging a more thoughtful and pragmatic approach to AI adoption [45].

Paradoxically, while AI is designed to enhance diagnostic efficiency, its implementation often increases the workload of physicians, fueling skepticism and reducing profitability. Instead of streamlining workflows and reallocating time to patient care, the physicians must invest time adapting to AI systems, troubleshooting errors, managing bureaucratic hurdles, and adjusting their routines. The time that was initially expected to be saved ultimately adds to their workload. Gawande [46] raised similar concerns, noting that the rigid implementation of technology frequently forces physicians to work overtime, resulting in frustration and uncertainties rather than freeing time for more meaningful tasks, such as patient interaction. This mismatch aligns with the law of Grudin [47], which posits that those required to use new technologies bear the burden of their inefficiencies. A notable example is the failed MD Anderson IBM Watson project, which was discontinued after costing more than US $62 million without ever having been integrated into clinical workflows, failing to meet expectations and resulting in wasted resources [48].

Beyond technical proficiency, the physicians expressed concern that an overreliance on AI could have severe consequences, potentially leading to worse patient outcomes. They warn that the tendency of AI to oversimplify complex cases [49] might undermine nuanced decision-making, increasing the risk of misdiagnoses or faulty treatments. More critically, excessive dependence on AI could diminish clinical judgment over time and create ambiguity in accountability when medical errors occur [50]. In addition, the physicians raised concerns about a potential skills gap. On the one hand, if AI becomes too embedded in daily practice without proper education or training, the physicians may either trust its outputs without critical evaluation or hesitate to use it because of a lack of confidence in its applications. On the other hand, if it becomes part of medical training, future physicians may not learn traditional hands-on skills as intensively, potentially weakening clinical skills over time. Despite these challenges, the interviewees expressed interest in interdisciplinary collaboration with AI, not as a replacement but as a complementary tool. However, a recent study indicates that effective collaboration between humans and AI remains elusive, especially in decision-making beyond image and text analysis [51]. While ongoing research explores AI agents as potential teammates in health care [52] or as providers of second opinions [53], their success depends on how they integrate without undermining the central expertise of physicians.

Most respondents (312/498, 62.7%) stated that they would consider themselves responsible for misdiagnoses involving AI, and 66.1% (329/498) of the respondents stated that they felt the same about faulty treatment decisions, a trend consistent with another study [31]. While physicians largely accept personal responsibility, the question of who is accountable when AI contributes to medical errors remains. This ambiguity underscores the need to clarify liability distribution among the physicians, AI developers, and institutions. Transparency emerged as an essential element of responsible AI integration. While most physicians did not demand complete transparency regarding the decision-making processes of AI, they insisted on receiving sufficient explanation to verify and critically evaluate AI-generated assessments. In this context, the physicians regarded AI as a complementary tool rather than a primary decision maker, underscoring the importance of maintaining human oversight [54].

The physicians acknowledged that AI, similar to humans, makes errors. Although some did not find this particularly concerning, recognizing that errors are inherent to medical practice, others expressed concern about an increased risk of bias, despite their generally positive perceptions [55]. The physicians were concerned that bias that are introduced by technology could be perpetuated if AI relies on repetitive patterns in existing databases. In radiology, for instance, cognitive bias may arise from highly repetitive pattern recognition tasks, such as analyzing large numbers of images daily, potentially affecting diagnostic accuracy [56]. In addition, racial bias has been reported in AI-based diagnostics [57].

Interestingly, contrary to common narratives [58], the physicians did not express fear of job replacement by AI. Some even welcomed changes to their traditional physician roles [59], viewing them as inevitable. The respondents considered AI as a tool for reassigning aspects of their work, allowing them to shift their focus from routine tasks toward more complex decision-making and patient-centered responsibilities. Within that role, the physicians may move toward a more managerial or coordinating function, overseeing AI-assisted systems. Concerns about role changes were mainly tied to limited AI knowledge, as shown in our study and other studies [30,44]. Amid physician shortages and high turnover, exacerbated by an aging workforce and rising retirements, AI could help to bridge health care gaps. However, despite their interest, many physicians lack the time, incentives, or resources to engage meaningfully with AI.

Despite the promise of increased efficiency and better patient care, the adoption of AI remains limited, with only 32.1% (160/498) of the physicians reporting that they use it in practice, consistent with the literature [20]. An equal proportion remains hesitant about the future use of AI, and 36.3% (181/498) report limited familiarity. Beyond familiarity, another major barrier is the lack of a robust infrastructure. Significant imbalances exist between public and private institutions regarding digital interconnectedness, infrastructure, and the pace of AI adoption. Private practices typically adopt new technologies faster, owing to fewer bureaucratic hurdles, greater financial flexibility, and profit-driven innovation. In contrast, public institutions often struggle with outdated IT systems, stand-alone technologies, and strict regulations, despite better access to research data and better interconnectivity [60-62]. Another key barrier is the lack of standardized and interoperable digital infrastructure, with data fragmented across incompatible systems, hindering seamless data exchange [15]. Many physicians noted that decentralized data storage creates inefficiencies, such as physically transferring patient records between institutions. However, some physicians viewed it as a safeguard against data security risks [57]. The adoption of AI is perceived as outpacing system readiness, leaving physicians frustrated with inefficient workflows and misaligned tools. Research shows that physicians often feel excluded from AI development processes [60], which lowers trust and hinders adoption [16]. To overcome barriers, AI must be user-friendly and integrated into medical education and training to bridge knowledge gaps. In addition, interdisciplinary collaboration on technical, regulatory, and operational levels would benefit implementation.

Notably, our survey did not find significant differences in attitudes toward AI based on age or years of experience. This contrasts with earlier studies suggesting that either younger [43] or older physicians [20] expressed more positive attitudes. However, we observed that chief physicians were more enthusiastic about the adoption of AI than residents [20], which may stem from the limited inclusion of AI-related topics in medical curricula. Although the broader literature on the adoption of technology suggests that older individuals are generally more resistant to digital transformation, this may not necessarily apply to clinical AI, where openness to collaboration and enthusiasm toward change may play a more decisive role than age alone [29]. Furthermore, our findings align with prior research indicating that attitudes toward AI do not vary across medical disciplines [41,42]. In addition, the attitudes toward the adoption of AI appeared to be context dependent [63]. For instance, anesthesiologists primarily framed their perspectives within the context of intensive care, possibly mirroring the perceived limited presence of AI in their field. At the same time, extensive research and development on AI exist in intensive care, including algorithm-optimized mechanical ventilation [64], on-site medical assistants, or visual speech recognition [65].

Finally, the physician-patient relationship remained central in shaping the perspectives of physicians toward the integration of AI. The physicians valued the potential of AI to increase diagnostic confidence, preventive capabilities, and therapeutic safety, ultimately facilitating more meaningful patient interactions and generally improving patient satisfaction. However, they expressed concern that increased patient access to generative AI tools (eg, ChatGPT and chatbots) might introduce misinformation into consultations [66]. AI promises to revolutionize how patients access medical information and treatment options. Consequently, there is a growing need to enhance AI competencies and AI literacy [43] among both patients and physicians.

### Limitations and Future Research

This study has some limitations. The participants lived or worked in Germany, limiting the generalizability beyond the national level. The recruitment methods may have introduced self-selection bias, as individuals with strong opinions on or experience with AI were more likely to participate. Most of the survey participants (303/498, 60.8%) were radiologists, likely because of support from the German Radiological Society in distributing the survey. Despite extensive recruitment efforts, including outreach through other medical societies, we did not achieve equally comparable group sizes across medical disciplines. This disciplinary imbalance may have led to an overrepresentation of perspectives from radiologists, some of whom may already engage with, for example, AI-based diagnostic tools in their practice, and an underrepresentation of fields, such as surgery or internal medicine, introducing response bias. Consequently, the quantitative findings may not fully represent the broader physician population. Therefore, we recommend that future studies use more balanced sampling approaches across medical disciplines. This could include methods such as quota sampling or outreach via a broader range of medical societies to better capture diverse and inclusive physician perspectives. There was also a slight gender imbalance in both the survey and the interviews. To account for uneven group sizes and the nonnormal distribution of survey data, we applied statistical methods that were less sensitive to group size differences, including the Kruskal-Wallis tests. Although no significant association was found between medical disciplines and attitudes toward AI, we encourage future research with balanced samples to confirm our findings. Given the use of convenience and purposive sampling, the study is subject to potential selection bias, which may limit the generalizability of the findings. However, these sampling strategies were selected to ensure the inclusion of participants with diverse perspectives and experiences regarding the adoption of AI in clinical practice. To address selection bias, we recruited participants through multiple medical networks, applied maximum variation sampling in interviews, and used methodological triangulation across quantitative and qualitative data sources. Nonetheless, the qualitative findings reflect a diversity of views, including those potentially underrepresented in the survey, providing depth of opinion that informed our propositions and recommendations. The survey is not a finalized measurement tool, and future iterations should refine and validate the attitudinal items to enhance reliability and applicability. The EFA identified 2 cross-loadings (ie, “open” and “skeptical”), which were retained because of their conceptual and thematic relevance. As with most qualitative research, findings are dependent on the specific time and context and should be interpreted accordingly. The time gap between the survey and the interviews was intentional and followed a sequential mixed methods approach, in which each method was analyzed separately [24]. Staggered data collection allowed the survey findings to inform the interview guide and facilitated a focused exploration of key themes. Although data collection occurred during a period of increased COVID-19 pandemic–driven digitalization and advancement of AI, the perspectives of physicians did not appear to change substantially. This likely reflects the slow integration of AI into German clinical practice, where its impact is not yet widely visible [67]. In addition, 2 interviews were conducted via phone rather than via video call, which may have influenced the depth of engagement or nonverbal communication. Moreover, we acknowledge that in qualitative research, the positionality of the researcher can shape how data are collected, interpreted, and understood. Therefore, we used investigator triangulation, engaged in interdisciplinary team discussions, and iteratively reflected on emerging findings to enhance critical reflexivity and analytic rigor. Finally, the views of medical students were not included. As they are among the first to experience the clinical uptake of AI fully, their insights could help tailor medical education programs and better prepare them for practice, as shown in previous research [68].

### Strengths and Implications

AI is no longer a distant future but actively shapes health care today. The 76% survey completion rate reflects strong interest, highlighting the relevance of AI and the importance of fostering engagement in research. To ensure that the adoption of AI does not create additional barriers, new workflows must emerge, and physicians must be equipped to work with AI effectively. Medical education programs and training courses across all career stages need to evolve and integrate digital health literacy and critical decision-making skills. Future engagement strategies should also target less experienced or research-distant physicians to bridge the gap in the adoption of AI. Despite much existing research, few AI applications reach clinical practice, often because of outdated workflows and infrastructure gaps. To avoid additional burdens and guarantee success, AI solutions must be seamlessly embedded into existing clinical processes and align with the needs of physicians. Our factor analysis revealed 2 key and contrasting dimensions of physician attitudes: AI enthusiasm and acceptance and AI skepticism and apprehension. These factors indicate the need for tailored strategies to guide implementation efforts and policy making. Physicians with greater enthusiasm and acceptance may be well suited for piloting new AI-based technologies, participating in peer training, or serving as mentors. In contrast, addressing skepticism and apprehension will require structured education, transparent communication, and clear accountability frameworks. Differentiated training programs and incentives aligned with these attitudinal profiles may help foster adoption while building trust. These results, together with our 6 established propositional statements and corresponding recommendations, can act as takeaways to inform future research and guide policy-making efforts.

### Conclusions

Physicians, regardless of discipline or age, view AI positively while expressing cautious skepticism. A higher level of personal and professional AI engagement—through familiarity, research involvement, personal or professional use, and willingness to use—is associated with both greater enthusiasm and acceptance, as well as less skepticism and apprehension. Men show a slightly more positive attitude than women, while chief physicians are significantly less skeptical than residents.

This study extends prior research by comprehensively assessing the attitudes and expectations of physicians toward the adoption of AI using a mixed methods approach. It highlights the need for broad engagement, targeted education and training, interoperable solutions, standardized guidelines, and seamless integration of AI into clinical workflows. By addressing these areas, physicians can benefit from AI, ensuring AI complements, rather than complicates, clinical practice.

## Appendix

Multimedia Appendix 1 This supplementary material includes the survey and interview guides, reporting checklists, a detailed breakdown of survey responses, and qualitative insights with illustrative comments.

## Abbreviations

AI: artificial intelligence
CHERRIES: Checklist for Reporting Results of Internet E-Surveys
EFA: exploratory factor analysis
