# Supplementary material for: Physicians’ Attitudes Toward Artificial Intelligence in Medicine: Mixed Methods Survey and Interview Study
Source: J Med Internet Res. 2025 Aug 26;27:e74187. doi: 10.2196/74187 (PMC12421205; doi:10.2196/74187)
Supplement: Multimedia Appendix 1 [file jmir_v27i1e74187_app1.pdf]

Multimedia Appendix 1

Physicians' Attitudes Toward Artificial Intelligence in Medicine: Mixed Methods Survey and Interview Study

Helen Heinrichs[1], MSc; Alexander Kies[2], Dr; Saskia K Nagel[3], Prof Dr; Fabian Kiessling[1,4,*], MD, Prof Dr

[1] Institute for Experimental Molecular Imaging, RWTH Aachen University, Aachen, Germany

[2] Service and Technology Marketing, RWTH Aachen University, Aachen, Germany

[3] Applied Ethics, RWTH Aachen University, Aachen, Germany

[4] Fraunhofer Institute for Digital Medicine MEVIS, Bremen, Germany

[*] Correspondence: fkiessling@ukaachen.de

Contents

Supplementary Material 1. Survey on the use of artificial intelligence in medicine among physicians.......... 3

Supplementary Material 2: Checklist for reporting results of internet e-surveys (CHERRIES). 8

Supplementary Material 3. Semi-structured interview guide on artificial intelligence in medicine among physicians. 11

Supplementary Material 4: Standards for reporting qualitative research (SRQR). 14

Table S1. Categorization of medical disciplines in the survey. 16

Table S2. Physicians' perspectives on AI in medicine: familiarity, future use, and accountability (N=498). . 17

Table S3. Expected advantages and disadvantages of AI in medicine (N=498). 18

Table S4. Physicians' perspectives on AI-induced changes in physician-patient communication (N=498).... 19

Table S5. Physicians' perspectives on AI in medical education, future impact, and training needs (N=498). 20

Table S6. Significance map of differences between Factor 1 and Factor 2 and demographic and professional characteristics. 21

Table S7. Differences between Factor 1 and Factor 2 and the panel of demographic and professional characteristics by groupwise and pairwise comparison. 22

Table S8. Coding categories of qualitative data, including subcategories, sample quotes and category descriptions. 24

Supplementary Material 1. Survey on the use of artificial intelligence in medicine among physicians.

Note: This survey has been translated from German into English. The translation was carried out with DeepL and reviewed by the research team.

Dear participant,
Welcome and thank you for your interest in our survey.
The ELSA-AID project is focused on analyzing ethical, social, and professional aspects that may change with the use of AI-supported diagnostics as part of precision medicine.
Artificial Intelligence (AI) refers to a machine taking on specific tasks or making decisions that would normally require human intelligence. In medicine, digitalization generates the necessary data for such machines to optimize or enable new treatment methods.
The digitalization of healthcare will continue to advance in the coming years. Opinions and attitudes about AI vary widely. Therefore, we aim to gather physicians' opinions on AI in medicine.
The questionnaire takes about 5 minutes to complete. The data will be collected anonymously and used solely for scientific research purposes.
The project, funded by the Federal Ministry of Education and Research, is a collaboration between RWTH Aachen, the University Hospital RWTH Aachen, and the Fraunhofer MEVIS Institute.

I hereby agree to the use and analysis of the data for scientific purposes in anonymized form.

- Yes
- No

-

Part 1: Personal Information

What is your gender?

- Woman
- Man
- Nonbinary

How old are you?

- < 30 years
- 30-39 years
- 40-49 years
- 50-59 years
- 60+ years

-

Your current position:

- Medical student
- Resident physician
- Attending physician (specialist)
- Attending physician (senior)
- Attending physician (chief)

Your medical specialty:

- General medicine
- Radiology
- Pathology
- Surgery
- Internal medicine
- Radiotherapy
- I am a resident physician
- I am a medical student
- Other: (please fill in)

How long have you been practicing your profession?

- I don't work yet (in medical school)
- < 10 years
- 10-20 years

- > 20 years

Do you work in research?
- Yes, in clinical research
- Yes, in experimental research
- Yes, in clinical and experimental research
- No

-

Are you involved in projects related to AI in medicine? (only applicable if the prior question was answered with yes)
- Yes
- No, but I can imagine doing so in the future
- No

-

Part 2: Opinions and attitudes

Are you generally interested in the topic of AI in medicine?
- Yes
- No
- Don't know

Do you use an AI-supported product in your private life?
Examples: Siri or Alexa as voice assistants, language translators such as Google Translate or DeepL, chatbots in customer service, car navigation, autonomous driving, etc.
- Yes [followed with an open text field: please specify]
- No, but I plan to do so in the future
- No
- Don't know

Do you use an AI-supported product in your daily clinical work?
Examples: image analysis of moles or X-ray images, image analysis in combination with other diagnostic data (e.g., genome data and laboratory parameters), nursing robots, etc.
- Yes [followed with an open text field: please specify]
- No, but I plan to do so in the future
- No
- Don't know

How familiar are you with the use of AI in medicine?
- Not at all familiar
- Somewhat familiar
- Moderately familiar
- Quite familiar
- Very familiar
- Don't know

How can you imagine AI being used in medicine in the future?
(Several answers possible)
- As an aid for medical staff
- As support in preventive diagnostics
- As a second opinion in decision-making
- As sole decision-maker
- Other: (please fill in)
- Don't know

-

How do you feel about the digital changes (including AI) in medicine?

	Strongly disagree	Disagree	Neither disagree nor agree	Agree	Strongly agree
Anxious	◦	◦	◦	◦	◦
Tense	◦	◦	◦	◦	◦
Skeptical	◦	◦	◦	◦	◦
Open	◦	◦	◦	◦	◦
Hopeful	◦	◦	◦	◦	◦
Curious	◦	◦	◦	◦	◦

-

Please indicate to what extent you agree or disagree with the following statements.

	Strongly disagree	Disagree	Neither disagree nor agree	Agree	Strongly agree	Don't know
AI can effectively support physicians.	◦	◦	◦	◦	◦	◦
The use of AI in medicine is superior to a physician's clinical experience.	◦	◦	◦	◦	◦	◦
I would seek a second opinion from an AI in decision-making processes.	◦	◦	◦	◦	◦	◦
The use of AI leads to a loss of control in my job.	◦	◦	◦	◦	◦	◦
Some medical specialties will be threatened by new technologies like AI in the future.	◦	◦	◦	◦	◦	◦
Some medical specialties will be enhanced by new technologies like AI in the future.	◦	◦	◦	◦	◦	◦
There are sufficient legal and technical-organizational guidelines for the use of AI in medicine.	◦	◦	◦	◦	◦	◦

Who should be responsible for incorrect diagnoses when AI is involved in decision-making?
(Several answers possible)

- Hospitals
- Medical device manufacturers
- Physicians
- It's patients' own risk
- Health insurance companies
- Other: (please fill in)
- Don't know

Who should be responsible for incorrect treatment decisions when AI is involved in the decision-making process?
(Several answers possible)

- Hospitals
- Medical device manufacturers
- Physicians
- It's patients' own risk
- Health insurance companies
- Other: (please fill in)
- Don't know

-

What advantages do you hope for from the use of AI in medicine?
(Several answers possible)

- More time for patients
- Less administrative tasks

- Relief during staff shortages
- Faster processing of routine tasks
- Increased diagnostic and therapeutic safety
- Faster diagnosis-making process
- Support in treatment decision-making
- Advanced drug research
- Execution of surgical procedures
- More personalized treatment
- Improved prevention (e.g., health assistants, enhanced screening)
- Automated diagnostic image analysis
- Other: (please fill in)
- I don't see any benefit
- Don't know

What disadvantages do you fear from the use of AI in medicine?
(Several answers possible)

- Reduced patient contact
- No guaranteed data protection
- Incomprehensible AI decision-making
- Undetected potential misdiagnoses
- Unclear liability in cases of potential misdiagnoses
- Overwhelm by new technology
- Other: (please fill in)
- I don't see any disadvantage
- Don't know

-

To what extent do you expect AI to change the communication between physicians and patients?

		Unchanged			Don't know
More impersonal	◦	◦	◦	More personal	◦
Less efficient	◦	◦		More efficient	◦
One-sided	◦	◦	◦	More interactive	◦
Less trustworthy	◦	◦	◦	More trustworthy	◦

The topic of AI in medicine is already sufficiently integrated into medical studies.

- Strongly disagree
- Disagree
- Neither disagree nor agree
- Agree
- Strongly agree
- Don't know

What should physicians be trained in regarding AI in medicine in the future?
(Several answers possible)

- Advantages and disadvantages of AI products
- Handling AI products
- Technical methods of AI (algorithms, etc.)
- Data protection
- Ethical questions about the use of AI
- Other: (please fill in)
- I don't see a need for training
- Don't know

At what speed do you expect AI to change your everyday working life fundamentally?
Within the next:

- 5 years
- 5-10 years
- 10-20 years

- > 20 years
- No change
- Don’t know

-

Comments (optional)
Is there anything else you would like to add to the topic of this survey? *[Open text box]*

Thank you for your participation! If you have any further questions or comments, please do not hesitate to contact us by e-mail.

Supplementary Material 2: Checklist for reporting results of internet e-surveys (CHERRIES).

**Item Category**	**Checklist Item**	**Explanation**	**Page Number**
Design	Describe survey design	Describe target population, sample frame. Is the sample a convenience sample? (In “open” surveys this is most likely.)	3
IRB approval and informed consent process	IRB approval	Mention whether the study has been approved by an IRB.	4
	Informed consent	Describe the informed consent process. Where were the participants told the length of time of the survey, which data were stored and where and for how long, who the investigator was, and the purpose of the study?	4
	Data protection	If any personal information was collected or stored, describe what mechanisms were used to protect unauthorized access.	N/A
Development and pre-testing	Development and testing	State how the survey was developed, including whether the usability and technical functionality of the electronic questionnaire had been tested before fielding the questionnaire.	3
Recruitment process and description of the sample	Open survey versus closed survey	An “open survey” is a survey open for each visitor of a site, while a closed survey is only open to a sample which the investigator knows (password-protected survey).	3
	Contact mode	Indicate whether or not the initial contact with the potential participants was made on the Internet. (Investigators may also send out questionnaires by mail and allow for Web-based data entry.)	3
	Advertising the survey	How/where was the survey announced or advertised? Some examples are offline media (newspapers), or online (mailing lists - If yes, which ones?) or banner ads (Where were these banner ads posted and what did they look like?). It is important to know the wording of the announcement as it will heavily influence who chooses to participate. Ideally the survey announcement should be published as an appendix.	3
Survey administration	Web/E-mail	State the type of e-survey (e.g., one posted on a Web site, or one sent out through e-mail). If it is an e-mail survey, were the responses entered manually into a database, or was there an automatic method for capturing responses?	3
	Context	Describe the Web site (for mailing list/newsgroup) in which the survey was posted. What is the Web site about, who is visiting it, what are visitors normally looking for? Discuss to what degree the content of the Web site could pre-select the sample or influence the results. For example, a survey about vaccination on a anti-immunization Web site will have different results from a Web survey conducted on a government Web site	N/A
	Mandatory/voluntary	Was it a mandatory survey to be filled in by every visitor who wanted to enter the Web site, or was it a voluntary survey?	3, 4
	Incentives	Were any incentives offered (e.g., monetary, prizes, or non-monetary incentives such as an offer to provide the survey results)?	N/A
	Time/Date	In what timeframe were the data collected?	3
	Randomization of items or questionnaires	To prevent biases items can be randomized or alternated.	N/A

	Adaptive questioning	Use adaptive questioning (certain items, or only conditionally displayed based on responses to other items) to reduce number and complexity of the questions.	3
	Number of Items	What was the number of questionnaire items per page? The number of items is an important factor for the completion rate.	SM1 [a]
	Number of screens (pages)	Over how many pages was the questionnaire distributed? The number of items is an important factor for the completion rate.	SM1 [a]
	Completeness check	It is technically possible to do consistency or completeness checks before the questionnaire is submitted. Was this done, and if “yes”, how (usually JAVAScript)? An alternative is to check for completeness after the questionnaire has been submitted (and highlight mandatory items). If this has been done, it should be reported. All items should provide a non-response option such as “not applicable” or “rather not say”, and selection of one response option should be enforced.	3
	Review step	State whether respondents were able to review and change their answers (e.g., through a Back button or a Review step which displays a summary of the responses and asks the respondents if they are correct).	3
Response rates	Unique site visitor	If you provide view rates or participation rates, you need to define how you determined a unique visitor. There are different techniques available, based on IP addresses or cookies or both.	N/A
	View rate (Ratio of unique survey visitors/unique site visitors)	Requires counting unique visitors to the first page of the survey, divided by the number of unique site visitors (not page views!). It is not unusual to have view rates of less than 0.1 % if the survey is voluntary.	N/A
	Participation rate (Ratio of unique visitors who agreed to participate/unique first survey page visitors)	Count the unique number of people who filled in the first survey page (or agreed to participate, for example by checking a checkbox), divided by visitors who visit the first page of the survey (or the informed consents page, if present). This can also be called “recruitment” rate.	N/A
	Completion rate (Ratio of users who finished the survey/users who agreed to participate)	The number of people submitting the last questionnaire page, divided by the number of people who agreed to participate (or submitted the first survey page). This is only relevant if there is a separate “informed consent” page or if the survey goes over several pages. This is a measure for attrition. Note that “completion” can involve leaving questionnaire items blank. This is not a measure for how completely questionnaires were filled in. (If you need a measure for this, use the word “completeness rate”.)	3
Preventing multiple entries from the same individual	Cookies used	Indicate whether cookies were used to assign a unique user identifier to each client computer. If so, mention the page on which the cookie was set and read, and how long the cookie was valid. Were duplicate entries avoided by preventing users access to the survey twice; or were duplicate database entries having the same user ID eliminated before analysis? In the latter case, which entries were kept for analysis (e.g., the first entry or the most recent)?	N/A
	IP check	Indicate whether the IP address of the client computer was used to identify potential duplicate entries from the same user. If so, mention the period of time for which no two entries from the same IP address were allowed (e.g., 24 hours). Were duplicate entries avoided by preventing	N/A

		users with the same IP address access to the survey twice; or were duplicate database entries having the same IP address within a given period of time eliminated before analysis? If the latter, which entries were kept for analysis (e.g., the first entry or the most recent)?	
	Log file analysis	Indicate whether other techniques to analyze the log file for identification of multiple entries were used. If so, please describe.	N/A
	Registration	In “closed” (non-open) surveys, users need to login first and it is easier to prevent duplicate entries from the same user. Describe how this was done. For example, was the survey never displayed a second time once the user had filled it in, or was the username stored together with the survey results and later eliminated? If the latter, which entries were kept for analysis (e.g., the first entry or the most recent)?	N/A
Analysis	Handling of incomplete questionnaires	Were only completed questionnaires analyzed? Were questionnaires which terminated early (where, for example, users did not go through all questionnaire pages) also analyzed?	3
	Questionnaires submitted with an atypical timestamp	Some investigators may measure the time people needed to fill in a questionnaire and exclude questionnaires that were submitted too soon. Specify the timeframe that was used as a cut-off point and describe how this point was determined.	3
	Statistical correction	Indicate whether any methods such as weighting of items or propensity scores have been used to adjust for the non-representative sample; if so, please describe the methods.	15

[a] SM is the abbreviation for Supplementary Material. From: Eysenbach, G. (2004). Improving the quality of Web surveys: the Checklist for Reporting Results of Internet E-Surveys (CHERRIES). *Journal of medical Internet research*, *6*(3), e34.

Supplementary Material 3. Semi-structured interview guide on artificial intelligence in medicine among physicians.

Note: This interview guide has been translated from German into English. The translation was carried out with DeepL and reviewed by the research team.

Start of the conservation:
We are interested in your personal perception, experience, and attitude to this topic. There are no right or wrong answers. Please be as detailed as possible in your answers.
As you have been informed through the consent form, all statements will be treated confidentially and anonymized. In order not to disrupt the conversation with handwritten notes and to make later evaluation easier, I would now like to start recording the audio of the conversation.

Category	Question	Follow-up Question
**Personal questions**		
	First, I would like to ask a few questions about yourself. Would you be so kind as to introduce yourself briefly?	
Personal questions	How old are you?	
	What is your medical specialty?	What is your position as a doctor?
	How long have you been practicing your profession?	
	Do you work in research?	<u>If yes:</u> Do you deal with AI-related topics within your research?
**Introduction**		
Icebreaker	To begin with, I would like to ask you: To what extent do new digital technologies play a role in your professional life?	<u>If positive:</u> What technologies do you use in your daily work?<br><br><u>If negative:</u> Is there a reason for this?
**Transition to the topic of AI**		
Approaching the topic of AI	To what extent have you knowingly gotten into contact with AI-based systems/tools in your professional context?	<u>If yes:</u> What application do you have in mind? Are you satisfied with this application? Are you missing certain functions?<br><br><u>If no:</u> What kind of possible applications could you imagine in your field?
Perception and current status	In which areas of your work do you particularly come into contact with AI applications?	In what situation? How would you describe the current status of AI applications in your work?<br><br><u>If there are no areas:</u> What comes to your mind spontaneously? How would you describe the current state of digitalization in your work?
**Main part**		
**Motivation and hesitation**		
Emotions	When you think about the use of AI in your field, does it evoke any emotions in you? If so, are they positive or negative?	<u>If positive:</u> Which emotions exactly? Which aspects of the use of AI evoke strong emotions in you?<br><br><u>If negative:</u> What concerns or doubts do you have? What negative feeling is evoked in you?<br><br><u>If hesitant:</u> Can you tell me how you experience the use of AI in your everyday work life?

Motivation	Do you see any short- or long-term developments as to how AI could impact your field?<br><br>Positively or negatively?	<u>If positive:</u> How would that impact your everyday work life?<br><br><u>If hesitant:</u> When you think about your everyday work, which task(s) could potentially be taken over by AI?
Restraint	What tasks or responsibilities do you think should not be transferred to AI, and why?	Are there any particular aspects that concern you? Are there any particular aspects that you think are too complex to be delegated to AI?
**Transparency and explainability**		
Diagnosis and therapy with AI	Can you imagine AI as a support in making diagnoses or in recommending therapy?	How should this support be designed? Could you please elaborate on that?
Perception diagnosis with AI	How would you react if you were confronted with a diagnosis/ therapy recommendation that was made using AI but contradicted your own findings?	How would you go about it?<br><br>In situations where the AI's conclusions conflict with other information or clinical judgment, what specific requirements must be met for you to fully understand its decision-making process?
Transparency/ explainability	How important is the transparency and explainability of the decision-making process of AI systems to you?	<u>If positive:</u> What level of understanding do you need for this? How deep do you think this understanding should be?<br><br><u>If negative:</u> Would a deeper understanding be helpful to you?
Liability	In our project, we also address legal issues: Does the allocation of liability play a decisive role for you to use AI? Or not to use it?	How do you think liability concerns could be effectively addressed?<br><br>Who should be responsible for the AI's decisions?
Perception moral responsibility	Some aspects of responsibility are regulated by law, while moral responsibility remains unaffected.<br><br>Philosophers in our team are discussing whether the increased use of AI will change the moral responsibility of physicians.<br>Do you think this will be the case, even if legal liability does not change for the time being?	Do you expect you will continue to perform treatments just as diligently when AI automates many of the standard processes?
**Human-machine interaction**		
Physician-patient communication	To what extent do you believe the increased use of AI will influence the interaction between physicians and patients? Positively or negatively?	<u>If positive:</u> What might that look like? In what context?<br><br><u>If negative:</u> How exactly?
	How do you perceive your colleagues' reactions to the use of AI? Is this a topic of discussion?	
**Needs and paint points**		
	Where do you see the greatest need for the use of AI?	How do you expect medical practice to change in the coming with the increased use of AI?
Needs	How comfortable or confident would you feel if AI were to be implemented tomorrow and you had to use this technology?	How would that make you feel?<br><br><u>If negative:</u> What would you need to feel secure?

Support	What do you wish for the integration process of AI in your work environment?	Do you have any ideas on how this process should be designed?<br><br>Can you name aspects that would be important to you?
Challenges	Where do you see challenge(s) related to AI integration?	
**Preparations for the changes**		
Readiness	How would you describe your current readiness for the upcoming changes?	Under what conditions would you be willing to accept AI?
Preparation	To what extent do you feel prepared for the upcoming changes?	<u>If positive:</u> How so? Have you taken any specific steps or measures to prepare yourself?<br><br><u>If negative:</u> Under what conditions would you feel prepared?
Support	To prepare for the use of AI in your work: Do you feel supported by your employer?<br><br>Self-employed: Are you already preparing for the use of AI?	Are you responsible for this yourself, or are there training programs or support measures offered by your employer?
Support/ training	What support measures would you personally need?	
Wishes	If you let your imagination run wild: What would you wish for AI in your everyday work?	
**Closing**		
Conclusion of the interview	Before we finish this interview, is there anything else you would like to add?	

Supplementary Material 4: Standards for reporting qualitative research (SRQR). [a]

Item Category	Checklist Item	Explanation	Page Number
Title and abstract	Title	Concise description of the nature and topic of the study Identifying the study as qualitative or indicating the approach (e.g., ethnography, grounded theory) or data collection methods (e.g., interview, focus group) is recommended.	1
	Abstract	Summary of key elements of the study using the abstract format of the intended publication; typically includes background, purpose, methods, results, and conclusions	1
Introduction	Problem formulation	Description and significance of the problem/phenomenon studied; review of relevant theory and empirical work; problem statement.	2
	Purpose or research question	Purpose of the study and specific objectives or questions.	2
Methods	Qualitative approach and research paradigm	Qualitative approach (e.g., ethnography, grounded theory, case study, phenomenology, narrative research) and guiding theory if appropriate; identifying the research paradigm (e.g., postpositivist, constructivist/ interpretivist) is also recommended; rationale [b].	3, 4
	Researcher characteristics and reflexivity	Researchers' characteristics that may influence the research, including personal attributes, qualifications/experience, relationship with participants, assumptions, and/or presuppositions; potential or actual interaction between researchers' characteristics and the research questions, approach, methods, results, and/or transferability.	3, 4, 15
	Context	Setting/site and salient contextual factors; rationale [b].	3, 4
	Sampling strategy	How and why research participants, documents, or events were selected; criteria for deciding when no further sampling was necessary (e.g., sampling saturation); rationale [b].	3, 4
	Ethical issues pertaining to human subjects	Documentation of approval by an appropriate ethics review board and participant consent, or explanation for lack thereof; other confidentiality and data security issues.	4
	Data collection methods	Types of data collected; details of data collection procedures including (as appropriate) start and stop dates of data collection and analysis, iterative process, triangulation of sources/methods, and modification of procedures in response to evolving study findings; rationale [b].	3, 4
	Data collection instruments and technologies	Description of instruments (e.g., interview guides, questionnaires) and devices (e.g., audio recorders) used for data collection; if/how the instrument(s) changed over the course of the study.	3, 4, SM3 [c]
	Units of study	Number and relevant characteristics of participants, documents, or events included in the study; level of participation (could be reported in results).	3, 4, 8, 9
	Data processing	Methods for processing data prior to and during analysis, including transcription, data entry, data management and security, verification of data integrity, data coding, and anonymization/de-identification of excerpts.	3, 4
	Data analysis	Process by which inferences, themes, etc., were identified and developed, including the researchers	3, 4, 8

		involved in data analysis; usually references a specific paradigm or approach; rationale [b].	
	Techniques to enhance trustworthiness	Techniques to enhance trustworthiness and credibility of data analysis (e.g., member checking, audit trail, triangulation); rationale [b].	3, 4
Results/ findings	Synthesis and interpretation	Main findings (e.g., interpretations, inferences, and themes); might include development of a theory or model, or integration with prior research or theory.	9-13
	Links to empirical data	Evidence (e.g., quotes, field notes, text excerpts, photographs) to substantiate analytic findings.	9-13
Discussion	Integration with prior work, implications, transferability, and contribution(s) to the field	Short summary of main findings; explanation of how findings and conclusions connect to, support, elaborate on, or challenge conclusions of earlier scholarship; discussion of scope of application/generalizability; identification of unique contribution(s) to scholarship in a discipline or field.	13-16
	Limitations	Trustworthiness and limitations of findings.	15
Other	Conflicts of interest	Potential sources of influence or perceived influence on study conduct and conclusions; how these were managed.	16
	Funding	Sources of funding and other support; role of funders in data collection, interpretation, and reporting.	16

[a] The authors created the SRQR by searching the literature to identify guidelines, reporting standards, and critical appraisal criteria for qualitative research; reviewing the reference lists of retrieved sources; and contacting experts to gain feedback. The SRQR aims to improve the transparency of all aspects of qualitative research by providing clear standards for reporting qualitative research. [b] The rationale should briefly discuss the justification for choosing that theory, approach, method, or technique rather than other options available, the assumptions and limitations implicit in those choices, and how those choices influence study conclusions and transferability. As appropriate, the rationale for several items might be discussed together. [c] SM is the abbreviation for Supplementary Material. From: O'Brien, B. C., Harris, I. B., Beckman, T. J., Reed, D. A., & Cook, D. A. (2014). Standards for reporting qualitative research: a synthesis of recommendations. *Academic medicine*, *89*(9), 1245-1251.

Table S1. Categorization of medical disciplines in the survey.

No.	Category names	Medical disciplines
1	Radiology	Radiology, diagnostic radiology, neuroradiology, pediatric radiology
2	Nuclear medicine & radiotherapy	Nuclear medicine, radiotherapy
3	Anesthesiology	Anesthesiology, anesthesia
4	Surgical disciplines	Surgery, neurosurgery, urology, oral and maxillofacial surgery, otorhinolaryngology, ophthalmology, dermatology, gynecology
5	Internal medicine	Internal medicine, general medicine, pediatrics, palliative medicine, cardiology
6	Others	Neurology, psychosomatic medicine, psychotherapy, pathology, laboratory medicine, human genetics, medical physics, residents

Notes: The initial categories of medical disciplines in the survey (see Supplementary Material 1) were adapted and categorized based on open-text answers and the frequency of answers provided. Most resident physicians specified their medical discipline; those residents who did not were classified as "Others".

Table S2. Physicians' perspectives on AI in medicine: familiarity, future use, and accountability (N=498).

Characteristic	n	% of physicians	% of answers
**How can you imagine AI being used in medicine in the future?**			***N*=1,246** [a]
Aid for medical staff	447	89.8	35.9
Support in preventive diagnostics	406	81.5	32.6
Second opinion in decision-making	312	62.7	25.0
Sole decision-maker	29	5.8	2.3
Other	48	9.6	3.9
Don't know	4	0.8	0.3
**Who should be responsible for incorrect diagnoses when AI is involved in decision-making?**			***N*=975** [a]
Hospitals	183	36.7	18.8
Medical device manufacturers	285	57.2	29.2
Physicians	312	62.7	32.0
Patients' own risk	49	9.8	5.0
Health insurance companies	56	11.2	5.7
Other	52	10.4	5.3
Don't know	38	7.6	3.9
**Who should be responsible for incorrect treatment decisions when AI is involved in decision-making?**			***N*=976** [a]
Hospitals	195	39.2	20.0
Medical device manufacturers	261	52.4	26.7
Physicians	329	66.1	33.7
Patients' own risk	45	9.0	4.6
Health insurance companies	59	11.8	6.0
Other	46	9.2	4.7
Don't know	41	8.2	4.2

[a] For multiple-response questions, percentages for N=498 show how many respondents selected an option, while percentages relating to the total number of answers indicate how often an option was selected across all responses. Since multiple responses were possible for these questions, the total number of answers exceeds the number of respondents.

Table S3. Expected advantages and disadvantages of AI in medicine (N=498).

Characteristic	n	% of physicians	% of answers
**Advantages**			***N*=3,237** [a]
More time for patients	264	53.0	8.2
Less administrative work	298	59.8	9.2
Relief during staff shortages	293	58.8	9.1
Faster processing of routine tasks	414	83.1	12.8
Increased diagnostic and therapeutic safety	373	74.9	11.5
Faster diagnosis-making process	245	49.2	7.6
Support in treatment decision-making	330	66.3	10.2
Advanced drug research	160	32.1	4.9
Execution of surgical procedures	40	8.0	1.2
More personalized treatment	198	39.8	6.1
Improved prevention (eg, health assistants, enhanced screening)	289	58.0	8.9
Automated diagnostic image analysis	312	62.7	9.6
Other	13	2.6	0.4
No benefits	6	1.2	0.2
Don’t know	2	0.4	0.1
**Disadvantages**			***N*=1,516** [a]
Reduced patient contact	111	22.3	7.3
No guaranteed data protection	118	23.7	7.8
Incomprehensible AI decision-making	377	75.7	24.9
Undetected potential misdiagnoses	295	59.2	19.5
Unclear liability in cases of potential misdiagnoses	411	82.5	27.1
Overwhelm by new technology	146	29.3	9.6
Other	43	8.6	2.8
No disadvantages	13	2.6	0.9
Don’t know	2	0.4	0.1

[a] For multiple-response questions, percentages for N=498 show how many respondents selected an option, while percentages relating to the total number of answers indicate how often an option was selected across all responses. Since multiple responses were possible for these questions, the total number of answers exceeds the number of respondents.

Table S4. Physicians' perspectives on AI-induced changes in physician-patient communication (N=498).

Characteristic	n	%
**To what extent do you expect AI to change the communication between physicians and patients?**		
More impersonal	108	21.7
Unchanged	263	52.8
More personal	80	16.1
Don't know	47	9.4
Less efficient	17	3.4
Unchanged	147	29.5
More efficient	292	58.6
Don't know	42	8.4
One-sided [a]	59	11.8
Unchanged	234	47.0
More interactive	158	31.7
Don't know	45	9.0
Less trustworthy	75	15.1
Unchanged	280	56.2
More trustworthy	86	17.3
Don't know	57	11.4

[a] Missing values are not shown as they account for ≤0.5% of the response distribution.

Table S5. Physicians' perspectives on AI in medical education, future impact, and training needs (N=498).

**Characteristic**	**n**	**% of physicians**	**% of answers**
**The topic of AI in medicine is already sufficiently integrated into medical studies.** [a]			
Strongly disagree	110	22.1	
Disagree	178	35.7	
Neutral	21	4.2	
Agree	15	3.0	
Strongly agree	5	1.0	
Don't know	168	33.7	
**At what speed do you expect AI to fundamentally change your everyday working life?**			
5 years	111	22.3	
5-10 years	252	50.6	
10-20 years	112	22.5	
> 20 years	10	2.0	
No change	9	1.8	
Don't know	4	0.8	
**What should physicians be trained on in the future regarding AI in medicine?**			***N*=1,961** [b]
Advantages and disadvantages of AI products	419	84.1	21.4
Handling of AI products	449	90.2	22.9
Technical methods of AI (algorithms, etc.)	355	71.3	18.1
Data protection	303	60.8	15.5
Ethical questions about AI use	407	81.7	20.8
Other	21	4.2	1.1
No need for training	2	0.4	0.1
Don't know	5	1.0	0.3

[a] Missing values are not shown as they account for ≤0.5% of the response distribution. [b] For multiple-response questions, percentages for N=498 show how many respondents selected an option, while percentages relating to the total number of answers indicate how often an option was selected across all responses. Since multiple responses were possible for these questions, the total number of answers exceeds the number of respondents.

Table S6. Significance map of differences between Factor 1 and Factor 2 and demographic and professional characteristics.

**Characteristics**	**Factor 1**	**Factor 2**
	***P*-value** [a]	***P*-value** [a]
Gender [b]	**.047**	.160
Age (years), categories	.212	.848
Medical disciplines	.389	.178
Physician's position	.107	**.023**
Professional experience (years), categories	.215	.174
Working in research [b]	**.003**	.170
Research related to AI	**<.001**	**.013**
Use of AI in everyday life	**<.001**	**.002**
Use of AI in professional context	**<.001**	**.001**
Familiarity with using AI	**<.001**	**.005**

[a] *P*-values indicate the significance level of differences in Factor 1 and Factor 2 across the listed characteristics (P<.05 bold). [b] The Mann-Whitney U test was used to compare two independent groups and the Kruskal-Wallis test was used to determine differences between three or more independent groups. Significant differences were compared in subgroups as a next step (see Supplementary Table 7).

Table S7. Differences between Factor 1 and Factor 2 and the panel of demographic and professional characteristics by groupwise and pairwise comparison.

Characteristics	Group Comparison	*n* [a]	Mean Ranks	Test Statistic (H or U) [b]	*P*-Value [d]
**Factor 1**					
Gender [c]	Men - Women	495	256.85-229.65	23932.500	.047
Working in research [c]	Yes, in clinical and experimental research - No	498	272.99-234.13	25021.000	.003
Research related to AI [e]	No - No, but I can imagine doing so in the future	96	54.32-95.20	40.878	**.009**
	No - Yes	123	54.32-111.52	57.202	**<.001**
	No, but I can imagine doing so in the future- Yes	175	95.20-111.52	16.324	.181
Use of AI in everyday life	No - Yes	465	165.76-260.71	94.949	**<.001**
	No - No, but I plan to do so in the future	105	165.76-295.45	129.693	**<.001**
	Yes - No, but I plan to do so in the future	424	260.71-295.45	-34.744	.561
Use of AI in professional context	No - Yes	342	196.17-253.88	57.711	**.001**
	No - No, but I plan to do so in the future	335	196.17-303.50	107.324	**<.001**
	Yes - No, but I plan to do so in the future	313	253.88-303.50	-49.612	**.006**
Familiarity with using AI	Not at all familiar - Moderately familiar	133	169.86-200.80	-30.939	1.000
	Not at all familiar - Somewhat familiar	181	169.86-236.72	-66.865	.124
	Not at all familiar - Quite familiar	210	169.86-279.82	-109.960	**<.001**
	Not at all familiar - Very familiar	74	169.86-323.55	-153.694	**<.001**
	Moderately familiar - Somewhat familiar	244	200.80-236.72	35.927	.527
	Moderately familiar - Quite familiar	273	200.80-279.82	-79.021	**<.001**
	Moderately familiar - Very familiar	137	200.80-323.55	-122.755	**<.001**
	Somewhat familiar - Quite familiar	321	236.72-279.82	-43.095	.068
	Somewhat familiar - Very familiar	185	236.72-323.55	-86.829	**.007**
	Quite familiar - Very familiar	214	279.82-323.55	-43.734	.820
**Factor 2**					
Physician's position	Resident - Specialist	238	210.60-245.30	-34.697	.519
	Resident - Senior	227	210.60-253.22	-42.615	.224
	Resident - Chief	177	210.60-277.32	-66.715	**.014**
	Specialist - Senior	321	245.30-253.22	-7.918	1.000
	Specialist - Chief	271	245.30-277.32	-32.018	.441
	Senior - Chief	260	253.22-277.32	-24.100	1.000

Research related to AI [e]	No - No, but I can imagine doing so in the future	96	70.45-94.83	24.377	.232
	No - Yes	123	70.45-108.27	37.818	**.014**
	No, but I can imagine doing so in the future - Yes	175	94.83-108.27	13.441	.366
Use of AI in everyday life	No - Yes	465	202.71-252.99	50.281	**.018**
	No - No, but I plan to do so in the future	105	202.71-305.67	102.960	**.002**
	Yes - No, but I plan to do so in the future	424	252.99-205.67	-52.678	.136
Use of AI in professional context	No - Yes	342	218.86-254.44	35.587	.064
	No - No, but I plan to do so in the future	335	218.86-275.93	57.071	**.001**
	Yes - No, but I plan to do so in the future	313	254.44-275.93	-21.484	.548
Familiarity with using AI	Not at all familiar - Moderately familiar	133	186.34-224.71	-38.371	1.000
	Not at all familiar - Somewhat familiar	181	186.34-259.58	-63.654	.155
	Not at all familiar - Quite familiar	210	186.34-250.00	-73.236	.061
	Not at all familiar - Very familiar	74	186.23-296.90	-110.555	**.008**
	Moderately familiar - Somewhat familiar	244	224.71-259.58	-25.283	1.000
	Moderately familiar - Quite familiar	273	224.71-250.00	34.864	.601
	Moderately familiar - Very familiar	137	224.71-296.90	-72.183	.073
	Somewhat familiar - Quite familiar	321	259.58-250.00	9.582	1.000
	Somewhat familiar - Very familiar	185	259.58-296.90	-46.900	.622
	Quite familiar - Very familiar	214	250.00-296.90	-37.319	1.000

[a] *n* represents the number of cases included in each particular comparison. [b] Kruskal-Wallis (H) and Mann-Whitney U test statistics measure rank distribution differences for multiple or [c] two-group comparisons, respectively. For multiple comparisons, Bonferroni-adjusted post-hoc tests were applied. [d] *P*-values indicate the significance level of differences in Factor 1 and Factor 2 across the listed groupwise comparisons ($P<.05$ bold). [e] Number of physicians engaged in research: $n=197$ (197/498).

Table S8. Coding categories of qualitative data, including subcategories, sample quotes and category descriptions.

(Sub-)categories [a]	Sample Quotes [b, c]	Description
**Status Quo**		
• Structural challenges<br>• Efficiency & AI relief<br>• Financial challenges	• "New systems take a very, very long time before they are installed; (...) several years, almost a decade." (IP2)<br>• "The only ones who can really expect anything from AI are the software companies entering the healthcare market and siphoning off limited resources." (SR670)	Status quo builds on interviewees' perceived current status of AI implementation in their professional lives. It includes views on practical, technological, and financial challenges as well as anticipated relief or hindrance from AI.
**AI Dependency & Negligence**		
• Dependency & bias<br>• Blind trust & negligence	• "Some people have automation bias, blindly following AI, while others intentionally oppose it, just to be against the AI." (IP8)<br>• "People might become overly dependent on AI, no longer questioning its outputs and blindly accepting results." (IP9)	This category refers to the risk of potentially losing critical skills due to physicians becoming overly dependent on or blindly trusting AI's outputs.
**Role Changes & Needs**		
• Medical experience<br>• Engagement & participation<br>• Medical training	• "Something new is introduced, you attend a single lecture, sign that you've been instructed, and you're expected to use it. But sorry, that's not how it works. (...) You need daily practical guidance." (IP9)<br>• "Many tasks may be replaced by AI, potentially preventing young colleagues from developing a deep understanding." (SR1397)	This category focuses on perceived role changes due to AI integration. It emphasizes maintaining human medical expertise, engaging physicians in the implementation of new technologies, and the need for ongoing training and support.
**AI Transparency & Decision-Making**		
• Complexity in decision-making<br>• Fallibility & trust	• "A crucial question is: Should checking the plausibility of AI outputs be my responsibility as a physician, or can I delegate it to someone who might even understand the process better than I do? This could allow me more time for direct patient care." (IP1)<br>• "If an AI error occurs, it would create significant public attention, similar to accidents with self-driving cars. There's still much work needed to achieve acceptance." (IP10)	Reflects the complexities associated with both human and AI-assisted decision-making, highlighting challenges related to transparency, fallibility, and trustworthiness of AI-assisted decisions.
**Physician-Patient Relationship**		
• Interaction & communication<br>• Patient information access	• "On one hand, this might create tension, but on the other hand, if it leads to a correct diagnosis and the right treatment, it could actually improve the physician-patient relationship—as long as the physician's pride isn't hurt." (IP10)<br>• "Nowadays, a patient with a rare condition can search their symptoms online and get a diagnosis. This certainly changes how patients approach us, and we must learn how to handle the probabilities generated by AI." (IP1)	Besides factors influencing physicians' interactions with new technologies, this category captures views on how AI impacts physician-patient interaction, communication, and patients' access to health information online.
**Framework for Responsible AI Integration**		

• Data security & legal framework<br>• Workflow compatibility	• "We are currently flooded with many immature and incompatible individual applications. Coordination and uniform standards would be desirable." (SR862)<br>• "AI shouldn't immediately take over everything in the hospital overnight; rather, it should be implemented gradually, based on proven success." (IP12)	Framework for responsible AI integration captures interviewees' expressed needs and recommendations for establishing regulations that ensure successful and ethical AI integration, particularly emphasizing data security, legal and liability considerations, and compatibility with existing workflows.

[a] Each category is further elaborated in the main text's results section, supported by additional illustrative quotes.
[b] Sample quotes represent data from qualitative interviews (IP) as well as from open-ended survey responses (SR).
[c] Sample quotes have been translated from German into American English.
